# Greenspace and Atopic Sensitization in Children and Adolescents—A Systematic Review

**DOI:** 10.3390/ijerph15112539

**Published:** 2018-11-13

**Authors:** Katrina A. Lambert, Gayan Bowatte, Rachel Tham, Caroline J. Lodge, Luke A. Prendergast, Joachim Heinrich, Michael J. Abramson, Shyamali C. Dharmage, Bircan Erbas

**Affiliations:** 1 School of Psychology and Public Health, La Trobe University, 3086 Bundoora, Australia; k.lambert@latrobe.edu.au (K.A.L.); b.erbas@latrobe.edu.au (B.E.); 2 Allergy and Lung Health Unit, Centre for Epidemiology and Biostatistics, Melbourne School of Population and Global Health, The University of Melbourne, 3000 Melbourne, Australia; gbowatte@gmail.com (G.B.); rachel.tham@unimelb.edu.au (R.T.); clodge@unimelb.edu.au (C.J.L.); heinrich@helmholtz-muenchen.de (J.H.); 3 National Institute of Fundamental Studies, Kandy 20000, Sri Lanka; 4 Behaviour, Environment and Cognition Research Program, Mary MacKillop Institute for Health Research, Australian Catholic University, 3065 Fitzroy, Australia; 5 Department of Mathematics and Statistics, La Trobe University, 3086 Bundoora, Australia; Luke.Prendergast@latrobe.edu.au; 6 Institute and Outpatient Clinic for Occupational, Social and Environmental Medicine, Clinical Center, Ludwig Maximilians University, Comprehensive Pneumology Centre Munich, German Centre for Lung Research, Ziemssenstrasse, 80336 Munich, Germany; 7 School of Public Health & Preventive Medicine, Monash University, 3004 Melbourne, Australia; michael.abramson@monash.edu

**Keywords:** atopy, IgE, skin prick, greenness

## Abstract

In the last decade, studies investigating greenspace have highlighted several benefits to human health. However, the effect of greenspace on allergies and atopic sensitization in children was not clear. While several studies have investigated this link, the evidence has not been systematically synthesized. We conducted a systematic search of eight databases. Study characteristics and findings were extracted from five articles covering 11 cohorts published between 2012 and 2016, and study quality assessments were performed. Due to significant heterogeneity, meta-analysis was not conducted. Findings were not consistent, possibly due to variations in exposure measurements, study populations and location, the specific allergens tested, and inclusion of confounders. Protective effects from greenspace were reported in four cohorts, while two cohorts showed an increase in sensitization related to greenspace. The other five cohorts found no significant effect of greenspace on atopic sensitization. There is limited understanding of the contributions of greenspace to specific allergens. Future research should consider amount and type of greenspace, as well as the specific allergens tested.

## 1. Introduction

The proportion of the world’s population living in urban areas is increasing sharply from just 30% in 1950 to a predicted 66% in 2050 [1]. The amount of contact with the natural environment in urban areas varies greatly within and between cities, with three quarters of the world’s cities having less green cover per person than World Health Organization (WHO) recommendations [2]. Limiting human contact with nature and biodiversity has the potential to seriously influence future population health [3]. Consequently, the effect of exposure to the natural environment in the form of greenness and greenspace on health outcomes has become an important factor to consider in environmental epidemiology.

In the last decade, greenspace has been shown to be beneficial to human health, particularly mental health [4], birth weight [5] and cardiovascular disease and all-cause mortality [6]. While not all research into these outcomes shows consistent effect [7], the literature as a whole supports a beneficial association between greenspace and a range of health outcomes.

The case for or against allergic disease is not as clear. A recent WHO report into urban greenspace [8] lists “risk of allergies and asthma” in a section entitled “Mechanisms of potential pathogenic effects of green spaces”. Studies in this area are contradictory, with our systematic review that examined associations with asthma and hay fever, finding both positive and negative associations in the literature for both outcomes [9].

While allergic diseases, including asthma, hay fever (allergic rhinitis) and other allergies, are increasingly common worldwide [10,11]. The burden of these diseases is high in pediatric populations and detrimentally affects school attendance and quality of life and increases health care use [12,13,14]. While there are multiple risk factors that have been investigated in the etiology of these conditions, except for second-hand smoke, hardly any modifiable factors have been established.

In this context, it is important to evaluate the role of greenspace in the pathway to allergic disease in children and adolescents. Atopic sensitization, the genetic propensity to develop immunoglobulin E (IgE) antibodies in response to exposure to food or inhalant allergens, can be objectively assessed either by measuring serum IgE levels in vitro or skin prick testing (SPT) and may provide insight into the pathway between greenspace and allergic disease [15]. The aim of this systematic review is to synthesize the current literature to assess whether surrounding greenspace is an important factor associated with atopic sensitization in children and adolescents.

## 2. Materials and Methods

### 2.1. Search Strategy

A systematic search of peer-reviewed literature following PRISMA guidelines was performed from inception up to 5 September 2018 using electronic bibliographic databases: AMED, CINAHL, Informit Health, Google Scholar, Medline, ProQuest central, Scopus and Web of Science. As greenspace can be measured in several different ways, an extensive list of search terms was used (Table S1). Further articles were found using citations from the publications included. This review was not prospectively registered.

### 2.2. Inclusion Criteria and Definitions

Population: Children and adolescents up to 19 years of age. Studies whose population was wholly within this range, had more than 90% of participants within this range, or stratified participants by age where at least one age band fell solely within this age range were included. 

Exposure: Any form of objective measure of greenspace, including vegetation levels, canopy cover, or measures of park, garden, or forest areas.

Comparison: No limits were set on comparison.

Outcome: Studies with an outcome of atopic sensitization to any allergen as measured by SPT or in vitro IgE measurements were included.

### 2.3. Selection of Included Articles

The abstracts of all identified papers were reviewed for initial inclusion by authors KL and GB; then full papers were read by KL to determine if all inclusion criteria were met. If KL was unsure of its merit for inclusion in the review the article was referred to GB for full paper review.

### 2.4. Data Extraction

Data extraction from included articles was performed in a standardized manner by KL and GB. Data were extracted from each article included: authors, year of publication, type of study, study population/country, number of children in the sample, age range, exposure definition, season of exposure measurement, outcome definition, risk estimates along with 95% confidence intervals (CI), confounders assessed, and any interactions assessed.

### 2.5. *Quality Assessment of the Included Studies*

The methodological quality of the studies included in this review were formally assess using a validated quality assessment framework [9]. This quality assessment tool provides evidence of the quality of a study based on factors such as the study sample, outcome and exposure assessment and adjustment of known confounders. Risk of bias in each individual study was categorized from none to high risk of bias. The authors resolved disagreements by consensus.

### 2.6. Assessment for Meta-Analysis

After assessing how the exposure and outcomes were measured in each study, no two studies provided effect size estimates for exposures measured in the same way as required for meta-analysis. Therefore, we were unable to conduct a meta-analysis.

## 3. Results

**Figure 1:**
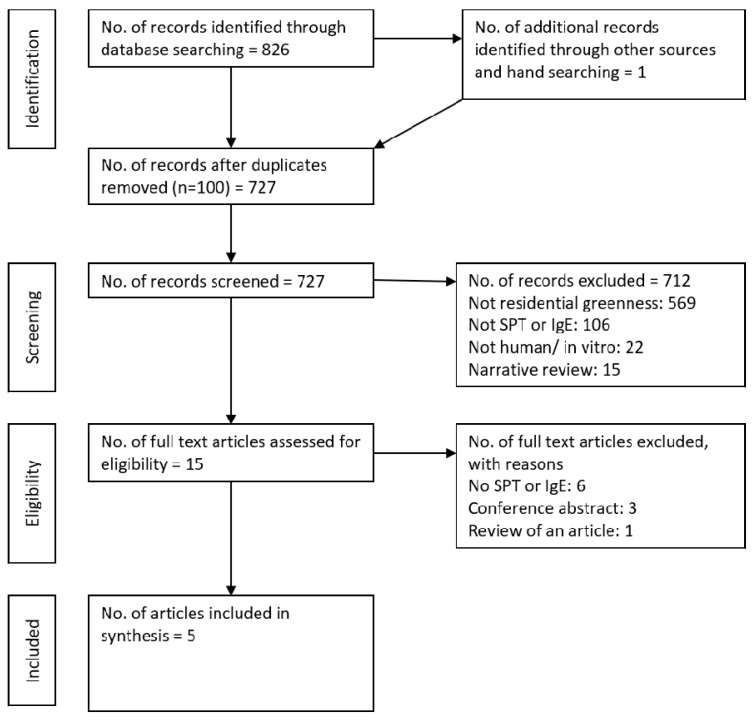
PRISMA flow diagram showing the progression of studies through the review.

Our search strategy identified 727 peer-reviewed articles after duplicate papers were removed (Figure 1). Of these, 712 were excluded following review of titles and abstracts. Of the remaining 15 articles, 10 were excluded following full-text assessment, for not including SPT or IgE, conference abstracts or including both children and adults, and five were identified for inclusion in this review.

### 3.1. Characteristics of Included Studies

The five papers included in this review report on 11 different cohorts, with three cohorts (KARA, GINIplus and LISAplus) being analyzed in more than one paper. The KARA cohort was used in two analyses [16,17], with Ruokolainen et al. [17] adding it to two other cohorts (LUKAS, DIABIMMUNE) to increase the statistical power to detect significant effects in their analysis. Two German cohorts (GINIplus and LISAplus) were analyzed twice; comparing North and South Germany [18] and included in a large study of seven cohorts [19]. The characteristics of the five papers are summarized in Table 1. Of these studies, population-based birth cohorts accounted for seven of the cohorts [17,18,19,20], 3 were high-risk cohorts (family history of atopic disease—exact definitions varied) (DIABIMMUNE, CAPPS, MACS) [17,19] and two studies included a random sample of school children assessed at two time points—aged 6 to 13 (2003) and aged 14 to 20 (2010) [16,17]. Most of the cohorts studied were based in Europe [16,17,18,19], with three cohorts in the US or Canada [19,20] and one in Australia [19].

### 3.2. Exposure Assessment

There were three different measurement techniques used to define greenspace, reflecting the different exposures. The Normalized Difference Vegetation Index (NDVI) used by two articles [18,19] is a measure of area-level vegetation intensity derived from publicly available satellite images. The NDVI is an index with values ranging between −1 and 1 and is calculated based on the ratio of visible (red) and near-infrared light reflection off the land surface [21]. The NDVI has been validated for use in epidemiological studies [22] and is recommended by the WHO as an indicator addressing the availability of overall greenness [8]. European specific land cover databases (CORINE 2000 and CORINE 2006) were used by two other articles [16,17]. These databases classify the dominant type of land cover into 44 classes with a minimum unit of 25 hectares. One article used a combination of high-resolution Light Detection and Ranging (LiDAR), color infrared aerial imagery and ancillary vector data to assess the proportions of tree canopy cover around the home [20].

### 3.3. Outcome Assessment

An atopic individual was defined by an increased in vitro specific IgE level to one or more aeroallergens in five of the 11 studied cohorts with a cut-off point of ≥0.35 kUA/L [18,19,20]. No studies looked at food allergens. General IgE was used as a measure of atopic sensitization in three cohorts with a cut-off of ≥2.5 kUA/L used for one cohort [16] and one article representing three cohorts [17] investigated multiple cut-off points (log10IgE = −0.5, 0, 0.5 and 1.0)—translating to approximately 0.32 kUA/L, 1 kUA/L, 3.16 kUA/L and 10 kUA/L on an arithmetic scale. In three of the cohorts (CAPS, MACS, SAGE) included in the study by Fuertes et al. [19] SPT was used instead of serum IgE, with a wheal size ≥3 mm to any allergens tested indicating an atopic individual.

The specific aeroallergens tested for in each cohort varied greatly (21 allergens combined), with the number of specific allergens tested varying from three in the MACS cohort [19] to 12 in the LUKAS cohort [17] with a median of 7 (Table S2). Only “cat dander” was tested in all 11 cohorts, although house dust mite (10), dog (9) and birch pollen (7) were also common. 

### 3.4. Quality Assessment and Risk of Bias

The overall methodological quality of the studies was high with most studies scoring >70% [18,19,20], despite the use of convenience sampling [20] or using multiple cohorts with different sampling methods [17,19]. The sample size of the KARA cohort [16,17] was small (*n* = 94) and neither study analyzing this cohort presented power calculations. One study [17] was rated low quality (<50%) due to the lack of reporting CIs and inconsistent reporting of sample size with no explanation. Only Fuertes and colleagues [18,19] adjusted for the potentially important environmental confounders of pollution and built environment. Overall, the risk of bias was low to moderate (Table S3).

### 3.5. Qualitative Synthesis of the Included Studies

The KARA cohort comes from a study in 2003 investigating differences between Finnish and Russian Karelia [23]. This study contained randomly selected schoolchildren, aged 6–16 years, and their mothers from Finland and Russia. Of the 546 child–mother pairs from Finland 344 children participated in SPT and IgE measurements. In 2010, Hanski and colleagues [16] followed up on 118 of these Finnish children now aged 14 to 18 years, again obtaining IgE measurements and quantifying the types of plants in the participants’ yards/gardens. They defined atopy based on IgE antibody level exceeding the cut-off value of 2.5 kUA/L for any allergen. Using principal component analysis and logistic regression of the data from 94 children with complete records, they found atopy decreased with an increase in the amount of forested and agricultural land within 3 km of the home as well as with an increase in flowering plants in the yard (Table 2). 

Multiple cut-off values for IgE where considered by Ruokolainen and colleagues [17], again using data from the KARA cohort (age 6–20 years, *n* = 94) as well as the DIABIMMUNE (age 0.5–3 years, *n* = 594) and LUKAS (age 1 and 6, *n* = 300) cohorts. The authors reported these cut-offs as expressed by log_10_(ΣIgE_inhalant_) > cut-off, with the logarithmic values of −0.5, 0, 0.5 and 1 translating to approximately 0.32 kU/L, 1 kU/L, 3.16 kU/L and 10 kU/L on an arithmetic scale. The relationship between the sum of the relative covers of forest and agricultural land within 3 km and atopy was non-significant in the DIABIMMUNE cohort regardless of the IgE cut-off used to define atopy. Increasing the proportions of forest and agricultural land in the LUKAS and KARA cohorts showed a systematic decline in the prevalence of atopy (exact numbers not presented).

The authors then pooled the data from the three cohorts into four different age groups: 0.5–1 years (DIABIMMUNE and LUKAS, *n* = 804), 1.5–3 years (DIABIMMUNE, *n* = 591), 6–12 years (LUKAS and KARA, *n* = 304), and 13–20 years (KARA, *n* = 95). The younger two age groups showed no significant relationship between land use and atopy, regardless of the cut-off used (*p*-values from 0.405 to 0.997). The 6–12 years showed a strong decrease in the prevalence of atopy at the thresholds of 0 (OR: 0.33, *p*-value: 0.034), 0.5 (OR: 0.17, *p*-value: 0.008) and 1 (OR: 0.21, *p*-value: 0.031). This relationship was not significant at the −0.5 threshold (OR: 0.42, *p*-value: 0.095). Conversely, the 13–20 years showed a similar strong decrease in the prevalence of atopy at all thresholds except the highest (−0.5 OR: 0.19, *p*-value: 0.023; 0 OR: 0.09, *p*-value: 0.003; 0.5, OR: 0.10, *p*-value: 0.009; 1, OR: 0.34, *p*-value: 0.229). No CIs were provided for further evaluation.

The LUKAS cohort was further used to investigate the “farming effect”. The farming effect is a phenomenon observed in studies from Europe, Canada, and Australia, which suggest that farming exposures offer protection against atopy in childhood [24]. Children living on farms in the LUKAS cohort were found to have significantly lower prevalence of atopy than children not living on farms overall (*p* = 0.032). However, when the proportion of forest and/ or agricultural land surrounding the home was included in the model as a land-use gradient, an independent farm effect was no longer significant. 

The Columbia Center for Children’s Environmental Health (CCCEH) birth cohort investigated a population of African American or Dominican children who lived in economically disadvantaged areas of New York City [20]. The authors based their analysis on the percentage of greenspace in the form of tree canopy cover around the home as measured by LiDAR related to aeroallergen sensitization measured by serum specific IgE using an IgE ≥ 0.35 kUA/L cut-off. They found an increase in greenspace within 250 m of the home was related to an increase risk of aeroallergen sensitization to any tested allergen after adjustment (RR: 1.20 95%CI: 1.05, 1.37). A stronger association was found between greenspace and allergic sensitization to tree pollen (RR: 1.43 95%CI: 1.19, 1.72).

A relationship between greenspace and allergic sensitization in the pooled GINIplus and LISAplus birth cohorts has been reported [18,19]. In both cases the pooled cohorts were split according to study region with GINI/LISA South covering participants from the city of Munich, Germany, and its surrounding areas and GINI/LISA North covering participants from the rural area near the former industrial Ruhr area in Germany. Greenspace was determined by NDVI at 500, 800, 1000 and 3000 m buffers around the birth, 6- and 10-year participant addresses and sensitization using serum IgE with a ≥0.35 kUA/L cut-off. The first report [18] presented results in terms of per interquartile (IQR) increase in mean greenness exposure and found increasing greenness was associated with reduced odds of aeroallergen sensitization in GINI/LISA North (OR: 0.78 95%CI: 0.65, 0.94) for 500 m at the ten-year address. This association was not present in GINI/LISA South (OR: 1.06 95%CI: 0.94, 1.20). While the results for other buffers and addresses were not presented, the authors stated them to be similar.

Fuertes and colleagues looked at the GINI/LISA data again in 2016 [19], this time presenting results in terms of a 0.2 increase in NDVI rather than IQR increase. Sensitization to any aeroallergen showed little association in either GINI/LISA North (age 6 OR: 0.79 95%CI: 0.56, 1.10; age 10 OR: 0.72 95%CI: 0.51, 1.02) or GINI/LISA South (age 6 OR: 1.15 95%CI: 0.90, 1.48; age 10 OR: 1.27 95%CI: 1.00, 1.60). However, greenspace was found to have a significant differential effect on sensitization to outdoor aeroallergens at age 10 with reduced odds in GINI/LISA North (OR: 0.63 95%CI: 0.43, 0.92) and increased odds in GINI/LISA South (OR: 1.45 95%CI: 1.13, 1.86).

Two other population-based birth cohorts considered by Fuertes et al. [19] used serum IgE to determine atopic sensitization. The BAMSE project comprised of over 4000 infants born in northern and central Stockholm, Sweden [25]. Aeroallergen sensitization in this cohort was measured at age 8 and showed increasing NDVI was related to increased odds of sensitization (OR: 1.41 95%CI: 1.15, 1.73). This association was consistent for both indoor (OR: 1.46 95%CI: 1.15, 1.86) and outdoor (OR: 1.44 95%CI: 1.15, 1.80) aeroallergens. Conversely, the Prevention and Incidence of Asthma and Mite Allergy (PIAMA) cohort from the Netherlands which was also tested at age 8 showed a non-significant reduction in the odds of aeroallergen sensitization (OR: 0.81 95%CI: 0.62, 1.05) which was stronger for sensitization to outdoor aeroallergens (OR: 0.72 95%CI: 0.53, 1.00). The PIAMA cohort was followed up at age 12, again showing a non-significant association between increasing NDVI and a reduction in the odds of aeroallergen sensitization (OR: 0.83 95%CI: 0.63, 1.09).

SPT was used to determine atopic sensitization in the Canadian Asthma Primary Prevention Study (CAPPS), the Melbourne Atopy Cohort Study (MACS) and the Study of Asthma, Genes, and Environment (SAGE) cohorts included in the 2016 study [19]. Sensitization in the high-risk CAPPS cohort was recorded at age 7 and, while non-significant overall (OR: 0.56 95%CI: 0.29, 1.06), increasing NDVI was associated with a reduction in odds of sensitization to outdoor aeroallergens (OR: 0.44 95%CI: 0.21, 0.93). The MACS cohort, also high risk, showed an overall reduction in odds of aeroallergen sensitization (OR: 0.57 95%CI: 0.34, 0.96) at age 12 that was related to indoor rather than outdoor aeroallergens. The SAGE cohort, based in Canada, measured aeroallergen sensitization at age 8 and found a difference between increase in NDVI and odds of indoor aeroallergen sensitization (OR: 0.69 95%CI: 0.46, 1.06) and outdoor aeroallergen sensitization (OR: 1.26 95%CI: 0.85, 1.86). 

### 3.6. Review by Exposure Methodology

Differences in the effect of residential greenness on atopy vary by exposure methodology (Table 3). Ruokolainen and colleagues [17] synthesized the studies using land-cover databases based on age and found a general reduction in odds of atopy, while the only study to use LiDAR [20] found a significant increase in the risk of atopy associated with greenness. NDVI was used most often and has disparate results. Fuertes and colleagues [19] pooled the results from the seven cohorts using NDVI, separating into childhood (6–8 years) and early adolescents (10–12 years) sensitization. The pooled results were highly heterogeneous (childhood: I^2^ = 73.8%, *p*-value = 0.0019; early adolescent: I^2^ = 76.5%, *p*-value = 0.0052) but not significant, overall. Stratification by sex, ambient nitrogen dioxide concentration, population density and dwelling in a rural/urban area in selected cohorts did not reveal any consistent patterns between cohorts.

## 4. Discussion

This is the first systematic review assessing the association between residential greenness and atopic sensitization in children and adolescents. We found five articles covering 11 study cohorts with three cohorts being studied more than once. Pooled results of multiple cohort studies were presented in two articles. Given the diverse outcome definitions and exposure assessments between studies a meta-analysis was not conducted. Protective effects were found in four of the cohorts (GINI/LISA North, KARA, LUKAS, MACS); while two cohorts (BAMSE, CCCEH) showed an increase in sensitization related to greenspace; and no association was found in five cohorts (CAPPS, DIABIMMUNE, GINI/LISA South, PIAMA, SAGE). Although these findings contribute to the development of a body of literature on residential greenness and atopic sensitization in children and adolescents the diverse findings as such make it difficult for conclusive interpretation of the effect sizes. 

Key issues in the studies were the assessment and definition of the outcome and age at measurement. The assessment and definition of atopic sensitization varied with some studies using SPT and others using serum IgE levels. The two methods of testing are not interchangeable, showing on average a 46 ± 24% agreement for aeroallergens - though this rate varies depending on specific allergen tested [26]. The number and type of specific allergens used in the testing varied substantially. The disparate results could be due, in part, to studies not detecting the same allergic sensitization. Differences in the age range of participants between studies further contributed to the heterogeneity, with ages at outcome ranging from six [18] to eighteen years old [16], not including the multi cohort study by Ruokolainen et al. [17] which included ages from six months to twenty years. The sample sizes investigated also varied significantly with the KARA cohort being quite small [16,17].

Another key issue was the exposure measurement. Two articles used NDVI as a measure of greenspace. NDVI is a useful measure to compare greenness globally as it is a standardized measure available at multiple time points. However, it is a crude measure that only distinguishes the amount of green foliage, not the types of vegetation. The CORINE land-cover database was used by two articles. The database only covers Europe and is not directly comparable to other land-cover databases due to differences in scale and categories, both in number and definition. Unlike NDVI which provides a standardized measure of greenness per pixel, land-cover databases are generally defined by the dominant land cover in an area and, depending on the scale, small areas of greenness can be overlooked. One article assessed greenspace by measuring the tree canopy using LiDAR, color infrared aerial imagery and ancillary vector data [20]. The size of buffer considered also varied greatly—from 250 m [20] to 3 km [17]. These factors create a diverse body of literature that cannot be easily synthesized. 

Due to the small number of studies investigating greenspace and atopy and the heterogeneity of exposures measured (NDVI, LiDAR, and land-cover databases), we were unable to conduct a meta-analysis. In addition, the timing of exposure varied greatly within studies with some considering the “birth” address or exposure measured within one or two years of the child’s birth, while others considered address at the time of the outcome measurement.

The methodological heterogeneity across the included studies as discussed is a potential driving cause of heterogeneous results. However, the study by Fuertes et al. [19] analyzed in parallel several birth cohorts, which were relatively homogenous in terms of exposure assessment, confounder control and statistical analysis. However, even these results were no more homogenous than the other included studies. Even within Germany and within one single study team, the results were very different for GINI/LISA North compared to GINI/LISA South. This suggest some residual confounding, perhaps by socio-economic status or residential address.

There is a sharp rural/urban divide in allergy prevalence [27] that is thought to be not just related to quantity but also quality of greenspace. The benefits of diverse wild vegetation compared to planned urban vegetation being a key component of the biodiversity hypothesis [28]. This dichotomy could be used to explain some of the differences in the literature. Ruokolainen et al. [17] and Hanski et al. [16], by virtue of using the CORINE land-cover data, had greenness that was areas of agricultural and forested land and excluded small scale greenspaces such as street trees and small urban parks/gardens. Whereas Lovasi et al. [20], by virtue of using LiDAR, a small buffer size and a study population in New York City, had greenness that was primarily street trees and urban parks/gardens. However, stratification of their cohort participants by a simple objective measure of urbanicity by Fuertes et al. [19] was non-significant overall despite a drastic reduction in the heterogeneity of the pooled estimates and BAMSE cohort reacting as expected. 

Allergic diseases are known, a least in part, to be hereditary [29]. As such, most cohorts attempted to adjust for potential confounding by genetic susceptibility including terms such as maternal asthma [20] and parental atopy [19]. No study reported on family history as a potential modifier of the association between greenness and atopic sensitization, although this was inherent in the study design of the “high-risk” cohorts. No study considered exposure to bioaerosols such as pollen and fungal spores which could also potentially modify the associations.

## 5. Conclusions

This is the first systematic review to qualitatively synthesize studies on greenspace and sensitization in children and adolescents. The findings show major inconsistencies in associations due to, but not limited to, study populations varying by age and type, exposure assessment, differences in type of allergens tested and lack of consideration of outdoor allergens as potential confounders and/or effect modifiers such as pollen and fungi. There is the very limited understanding of the impact of greenspace and allergies and particularly the underlying mechanisms. Future studies should focus on the gaps identified through this review, especially developing international consensus on how best to measure the greenness to improve the quality of the research and focus on understanding the underlying mechanisms.

## Figures and Tables

**Table 1:** Characteristics of included studies.

Author (Year)	Country (Cohort)	*n*	Study Design	Exposure Metric	Address at Age	Outcome (Cut-Off)	Age at Outcome	Risk of Bias
Hanski et al. (2012)	Finland (KARA)	94	Cross sectional sample with follow up	Land-cover database	14 to 18	IgE (≥2.5 kU/L)	14 to 18	Mod
Lovasi et al. (2013)	USA (CCCEH)	549	Population-based birth cohort	LiDAR imagery	Prenatal (sensitivity using address at age 7)	IgE (≥0.35 kU/L)	7	Low
Fuertes et al. (2014b)	Germany (GINIplus and LISAplus combined)	5803	Population-based birth cohorts	NDVI	Birth, 6- and 10-year addresses	IgE (≥0.35 kU/L)	6 and 10	Low
Ruokolainen et al. (2015)	Finland (KARA)	94	Cross sectional sample with follow up	Land-cover database	Not stated	IgE (multiple)	6 to 20	Mod
	Finland (LUKAS)	300	Population-based birth cohorts		Birth	IgE (multiple)	1 and 6	
	Estonia and Finland (DIABIMMUNE)	594	High-Risk birth cohorts		Birth	IgE (multiple)	0.5, 1.5 and 3	
Fuertes et al. (2016)	Sweden (BAMSE)	13,016	Population-based birth cohort	NDVI	6 to 8 10 to 12	IgE (≥0.35 kU/L)	6 to 8 10 to 12	Low
	Canada (CAPPS)		Randomized controlled study with asthma intervention			SPT (≥3 mm)		
	Germany (GINIplus and LISAplus combined)		Population-based birth cohorts			IgE (≥0.35 kU/L)		
	Australia (MACS)		High-Risk birth cohort			SPT (≥3 mm)		
	Netherlands (PIAMA)		Population-based birth cohort			IgE (≥0.35 kU/L)		
	Canada (SAGE)		Population-based birth cohort			SPT (≥3 mm)		

NDVI: Normalized Difference Vegetation Index; LiDAR: Light Detection and Ranging; SPT: Skin Prick Testing; IgE: allergen-specific Immunoglobin E level.

**Table 2:** Detailed characteristics of studies examining effect of residential greenness on outcomes related to atopic sensation.

Author (Year)	Exposure Definition	Outcome Definition	Exposure Estimate (95% CI)
Hanski et al. (2012)	Vegetation cover of the yards and the major land-use types within 3 km of the homes of the study subjects.	Atopic individuals based on IgE antibody level with a cut-off value of 2.5 kU_A_/L	Increase in one unit of first principal component of land-use types OR: 0.594 (no 95%CI reported) Increase in the number of uncommon native flowering plant species in the yard OR: 0.905 (no 95%CI reported)
Lovasi et al. (2013)	Urban tree canopy coverage (combined high-resolution Light Detection and Ranging (LiDAR) data and color infrared aerial imagery) for address at time of birth (250 m).	Serum IgE antibody level with a cut-off value of 0.35 IU/mL	Relative risk (RR) increase per standard deviation of tree canopy coverage 1.20 (1.05, 1.37)
Fuertes et al. (2014b)	Residential greenness in a 500 m buffer around the 10-year home addresses (NDVI)	Aeroallergen sensitization (at 6 and 10 years)—IgE ≥ 0.35 kU/L	Aeroallergen sensitization (OR) per increase in mean NDVI: 0.96 (0.85, 1.07)
Ruokolainen et al. (2015)	The coverage of five land-use types (agricultural land, built area, forest, water bodies and wetland) around each home was calculated with the CORINE2006 land-cover data using a buffer with radius of 3 km.	Atopy (atopic sensitization) was defined based on the sum of IgE antibodies that are specific to inhalant allergens, such that an individual with log_10_(ΣIgE*_inhalant_*) > IgE*_th_* was classified as atopic, where IgE*_th_* is a cut-off level.	Significant (*p*-value < 0.05) associations were found for all log_10_ cut-off values (IgE*_th_* = −0.5 OR: 0.61 IgE*_th_* = 0 OR: 0.42, IgE*_th_* = 0.5 OR: 0.24, IgE*_th_* = 1 OR: 0.30). 95%CI were not reported.
Fuertes et al. (2016)	Mean NDVI at 500m and 1000m circular buffers around home address (age 6–8 and 10–12) taken during the spring and summer months of year of birth.	Sensitization was assessed by skin prick testing for CAPPS, MACS, and SAGE, with a positive reaction defined as having a wheal diameter of ≥3 mm. For all other cohorts, sensitization was assessed by measuring allergen-specific IgE levels, with a positive reaction defined as any value ≥0.35 kU/L	OR per 0.2 unit increase in mean NDVI Age 6–8: BAMSE: 1.41 (1.15, 1.73) CAPPS: 0.56 (0.29, 1.06) GINI/LISA North: 0.79 (0.56, 1.10) GINI/LISA South: 1.15 (0.90, 1.48) PIAMA: 0.81 (0.62, 1.05) SAGE: 0.93 (0.65, 1.32) Pooled Result: 0.96 (0.75, 1.22) Age 10–12: GINI/LISA North: 0.72 (0.51, 1.02) GINI/LISA South: 1.27 (1.00, 1.60) MACS: 0.57 (0.34, 0.96) PIAMA: 0.83 (0.63, 1.09) Pooled Result: 0.85 (0.61, 1.18)

NDVI: Normalized Difference Vegetation Index; LiDAR: Light Detection and Ranging.

**Table 3:** Results by exposure methodology.

Exposure Measure	Number of Cohorts	Distance	Age at Outcome	Name	Result	Note
**Land-cover database**	3	3 km	0.5–1 years old	DIABIMMUNE and LUKAS (pooled)	OR: 1.00 *p* = 0.997	Atopy defined as >1 kU/L total IgE. No confidence intervals provided
			1.5–3 years old	DIABIMMUNE	OR: 0.83 *p* = 0.693	
			6–12 years old	LUKAS and KARA (pooled)	OR: 0.33 *p* = 0.034	
			13–20 years old	KARA	OR: 0.09 *p* = 0.003	
**LiDAR imagery**	1	250 m	7 years old	CCCEH	RR: 1.20 (1.05, 1.37)	
**NDVI**	7	500 m	6–8 years old	BAMSE	OR: 1.41 (1.15, 1.73)	
				CAPPS	OR: 0.56 (0.29, 1.06)	
				GINI/LISA North	OR: 0.79 (0.56, 1.10)	
				GINI/LISA South	OR: 1.15 (0.90, 1.48)	
				PIAMA	OR: 0.81 (0.62, 1.05)	
				SAGE	OR: 0.93 (0.65, 1.32)	
				Pooled Result *	OR: 0.96 (0.75, 1.22)	Heterogeneity: I^2^ = 73.8% *p* = 0.0019
			10–12 years old	GINI/LISA North	OR: 0.72 (0.51, 1.02)	
				GINI/LISA South	OR: 1.27 (1.00, 1.60)	
				MACS	OR: 0.57 (0.34, 0.96)	
				PIAMA	OR: 0.83 (0.63, 1.09)	
				Pooled Results *	OR: 0.85 (0.61, 1.18)	Heterogeneity: I^2^ = 76.5% *p* = 0.0052

* Pooled results from Fuertes et al. [19]. No original analysis conducted.

## Supplementary Materials

The following are available online at http://www.mdpi.com/1660-4601/15/11/2539/s1. Table S1: Ovid search strategy for initial literature review, Table S2: Specific aeroallergens tested for in each cohort, Table S3: Bias assessment.
