# Supplementary material for: Greenspace and Atopic Sensitization in Children and Adolescents—A Systematic Review
Source: Int J Environ Res Public Health. 2018 Nov 13;15(11):2539. doi: 10.3390/ijerph15112539 (PMC6265981; doi:10.3390/ijerph15112539)
Supplement: Supplementary file 1 [file ijerph-15-02539-s001.pdf]

Table S1: Ovid search strategy for initial literature review

1	“allergic sen*”
2	atopy
3	atopic
4	Hypersensitivity
5	“Skin Prick”
6	IgE
7	“Immunoglobulin E”
8	Sensiti?ation
9	1 or 2 or 3 or 4 or 5 or 6 or 7 or 8
10	NDVI
11	“Normali?ed Difference Vegetation Index”
12	EVI
13	“Enhanced Vegetation Index”
14	“Leaf Area Index”
15	biosphere
16	“residential green*”
17	“green space”
18	greenness
19	“tree canopy”
20	“tree cover”
21	vegetation
22	greenspace
23	10 or 11 or 12 or 13 or 14 or 15 or 16 or 17 or 18 or 19 or 20 or 21 or 22
23	9 AND 23

("allergic sen*" OR atopy OR atopic OR Hypersensitivity OR IgE OR "Immunoglobulin E" OR Sensiti?ation) AND (NDVI OR "Normali?ed Difference Vegetation Index" OR "Enhanced Vegetation Index" OR "residential green*" OR "green space")

Table S2: Specific aeroallergens tested for in each cohort

	Ruokolainen et al. 2015				Fuertes et al. 2016						
	Hanski et al. 2012			Lovasi et al. 2013	Fuertes et al. 2014b						
	KARA	DIABIMMUNE	LUKAS	CCCEH	GINIplus	LISAplus	BAMSE	CAPPS	MACS	PIAMA	SAGE
Alder			x								
Alternaria			x					x		x	
Birch	x	x	x		x	x	x			x	
Cat	x	x	x	x	x	x	x	x	x	x	x
Cockroach				x				x			
Dactylis glomerata										x	
Dog	x	x	x	x		x	x	x		x	x
European Hazel			x								
Feathers								x			x
'Grass'			x	x				x			x
Horse	x		x								
House dust mite	x	x	x	x	x	x	x	x	x	x	
Mold	x			x	x	x	x	x			
Mouse				x							
Mugwort	x		x		x	x	x				
Plantain			x								
ragweed				x				x			x
Rye			x		x	x			x		
Timothy grass	x	x			x	x	x				
'Trees'				x				x			x
'Weeds'								x			x
Total number of allergens tested	8	5	12	9	7	8	7	11	3	6	7

Table S3: Bias assessment

Author (Year)	Exposure definition bias	Exposure misclassification bias	Selection bias	Ascertainment bias	Confounding bias	Confidence intervals
Hanski I, von Hertzen L. (2012)						
Lovasi, GS, O'Neil-Dunne, JPM et al. (2013)						
Fuertes, E, Markevych, I, et al. (2014)						
Ruokolainen L, von Hertzen L, Fyhrquist N. (2015)						
Fuertes, E., I. Markevych, et al. (2016).						

No risk of bias   Low risk of bias   Moderate risk of bias   High risk of bias
